# Regional differences in antihyperglycemic medication are not explained by individual socioeconomic status, regional deprivation, and regional health care services. Observational results from the German DIAB-CORE consortium

**DOI:** 10.1371/journal.pone.0191559

**Published:** 2018-01-25

**Authors:** Christina Bächle, Heiner Claessen, Werner Maier, Teresa Tamayo, Michaela Schunk, Ina-Maria Rückert-Eheberg, Rolf Holle, Christa Meisinger, Susanne Moebus, Karl-Heinz Jöckel, Sabine Schipf, Henry Völzke, Saskia Hartwig, Alexander Kluttig, Lars Kroll, Ute Linnenkamp, Andrea Icks

**Affiliations:** 1 Institute for Health Services Research and Health Economics, German Diabetes Centre, Leibniz Center for Diabetes Research at Heinrich-Heine-University, Düsseldorf, Germany; 2 German Center for Diabetes Research (DZD), München-Neuherberg, Germany; 3 Institute of Health Economics and Health Care Management, Helmholtz Zentrum München, German Research Center for Environmental Health, Neuherberg, Germany; 4 Institute of Epidemiology II, Helmholtz Zentrum München, German Research Center for Environmental Health, Neuherberg, Germany; 5 Institute for Medical Informatics, Biometry and Epidemiology, University Hospital of Essen, University Duisburg-Essen, Germany; 6 Institute for Community Medicine, University Medicine Greifswald, Greifswald, Germany; 7 Institute of Medical Epidemiology, Biostatistics and Informatics, Martin-Luther-University Halle-Wittenberg, Halle (Saale), Germany; 8 Department of Epidemiology and Health Monitoring, Robert Koch Institute, Berlin, Germany; 9 Institute for Health Services Research and Health Economics, Faculty of Medicine, Heinrich Heine University Düsseldorf, Düsseldorf, Germany; Medizinische Universitat Innsbruck, AUSTRIA

## Abstract

**Aims:**

This population-based study sought to extend knowledge on factors explaining regional differences in type 2 diabetes mellitus medication patterns in Germany.

**Methods:**

Individual baseline and follow-up data from four regional population-based German cohort studies (SHIP [northeast], CARLA [east], HNR [west], KORA [south]) conducted between 1997 and 2010 were pooled and merged with both data on regional deprivation and regional health care services. To analyze regional differences in any or newer anti-hyperglycemic medication, medication prevalence ratios (PRs) were estimated using multivariable Poisson regression models with a robust error variance adjusted gradually for individual and regional variables.

**Results:**

The study population consisted of 1,437 people aged 45 to 74 years at baseline, (corresponding to 49 to 83 years at follow-up) with self-reported type 2 diabetes. The prevalence of receiving any anti-hyperglycemic medication was 16% higher in KORA (PR 1.16 [1.08–1.25]), 10% higher in CARLA (1.10 [1.01–1.18]), and 7% higher in SHIP (PR 1.07 [1.00–1.15]) than in HNR. The prevalence of receiving newer anti-hyperglycemic medication was 49% higher in KORA (1.49 [1.09–2.05]), 41% higher in CARLA (1.41 [1.02–1.96]) and 1% higher in SHIP (1.01 [0.72–1.41]) than in HNR, respectively. After gradual adjustment for individual variables, regional deprivation and health care services, the effects only changed slightly.

**Conclusions:**

Neither comprehensive individual factors including socioeconomic status nor regional deprivation or indicators of regional health care services were able to sufficiently explain regional differences in anti-hyperglycemic treatment in Germany. To understand the underlying causes, further research is needed.

## 1. Introduction

Diabetes has been proclaimed to be one of the most challenging health problems of the 21^st^ century [1]. In a Germany-wide survey, the prevalence of known type 2 diabetes mellitus was estimated to be 7.2% in 2012 [2]. However, regional prevalence estimates showed a southwest-to-northeast-gradient of type 2 diabetes prevalence with the lowest prevalence in the south (KORA S4; 5.8%) and the highest estimates in the east (CARLA, 12.0%) [3]. As revealed in further analyses, regional differences in type 2 diabetes mellitus prevalence were not solely attributable to individual characteristics: regional deprivation on municipality and district level as well as neighborhood unemployment rate turned out to influence type 2 diabetes prevalence independently [4–7].

Regional differences were also observed in terms of type 2 diabetes mellitus therapy and outcomes and regional deprivation turned out to be an additional independent factor of growing importance for health care utilization and outcomes [8]. In a systematic review summarizing the results of 21 studies published between January 2002 and December 2011, Grintsova et al. pointed out that people with low socioeconomic status (SES) tended to receive worse diabetes care (e.g. low frequency of HbA1c measurement) and have poorer intermediate diabetes outcomes [8]. Living in deprived areas was associated with less frequent achievement of glycemic control targets, a trend towards higher blood pressure and worse lipid profile control. These results were confirmed by a recently published population based study from North Karelia Finland [9].

Regional differences in Medicare reimbursement per patient in 2014 were found in the U.S. with almost two-fold higher expenditures in Florida compared to Alaska [10]. In Germany, Schipf et al. analyzed the regional prevalence of anti-hyperglycemic medication [3] among participants from five population-based studies and found variations between 75.4% (HNR baseline study) and 86.3% (KORA S4). To explain these differences, another study investigated associations with participants’ individual characteristics including socioeconomic status [11] based on data from two of these regional studies (KORA F4, HNR follow-up study). However, despite considering a wide selection of covariates, among them education, body mass index, blood pressure, comorbidity, health insurance status, family status, and lifestyle measures, regional differences in any and newer antihyperglycemic medication (mainly introduced around the year 2000) could not be explained.

The aim of this study was to extend knowledge on factors explaining regional antihyperglycemic medication patterns. Therefore, individual baseline and follow-up data from four regional population-based studies in Germany were analyzed and complemented by the German Index of Multiple Deprivation [4,5] and indicators of regional health care services.

## 2. Research design and methods

The current study is based on the study methods and contents of Tamayo et al. [11] adding further study regions, significantly increasing the study population, and extending the study period.

### 2.1 Data sources and description of variables

#### 2.1.1 Regional studies and study population

Baseline and follow-up data from four regional population-based cohort studies carried out in Germany were included (Table 1).

**Table 1:** Included diabetes collaborative research of epidemiologic studies (DIAB-CORE).

Study	Region	Baseline examination	N_baseline_ (N type 2 diabetes mellitus)^a^	follow-up examination	N_follow-up_ (N type 2 diabetes mellitus)^a^
The Study of Health in Pomerania (SHIP):	Mecklenburg West Pomerania (northeast)	1997–2001	2,277 (251)	2002–2006	1,828 (287)
The Cardiovascular Disease, Living and Ageing in Halle Study (CARLA)	City of Halle, Saxony-Anhalt (east)	2002–2006	1,417 (174)	2007–2010	1,211 (198)
The Heinz Nixdorf Recall Study (HNR)	Cities of Essen, Bochum and Mülheim (Ruhr area), North Rhine-Westphalia (west)	2000–2003	4,814 (348)	2006–2008	4,157 (493)
The Cooperative Health Research in the Region of Augsburg Survey 4 (KORA S4)	City of Augsburg and two surrounding rural areas, Bavaria (south)	1999–2001	2,524 (146)	2006–2008	1,862 (195)
**Total study population**			**11,032 (919)**		**9,058 (1,173)**

^a^ age group 45 to 74 years at baseline examination (49 to 83 years at follow up examination)

The four studies are comparable in their study design, sampling methods, study population, and response rates (baseline 56%-69%, follow-up 80%-90%). Study details have been described previously [12–16]. All studies weremonitored by independent scientific advisory boards. All participants gave their written consent.

All study methods were approved by the Ethics Committee of the Medical Faculty of the Martin-Luther-University Halle-Wittenberg and by the State Data Privacy Commissioner of Saxony-Anhalt (CARLA), by the Medical Ethics Committee of the University of Greifswald (SHIP), by the Ethics Committee of the Bavarian Medical Association (KORA) and by the institutional local ethical committees (baseline: Medical faculty University of Essen; follow-up: Medical faculty University of Duisburg-Essen) (Heinz Nixdorf Recall (HNR). Primary study data of interest were pooled and frequencies compared.

**Fig 1:**
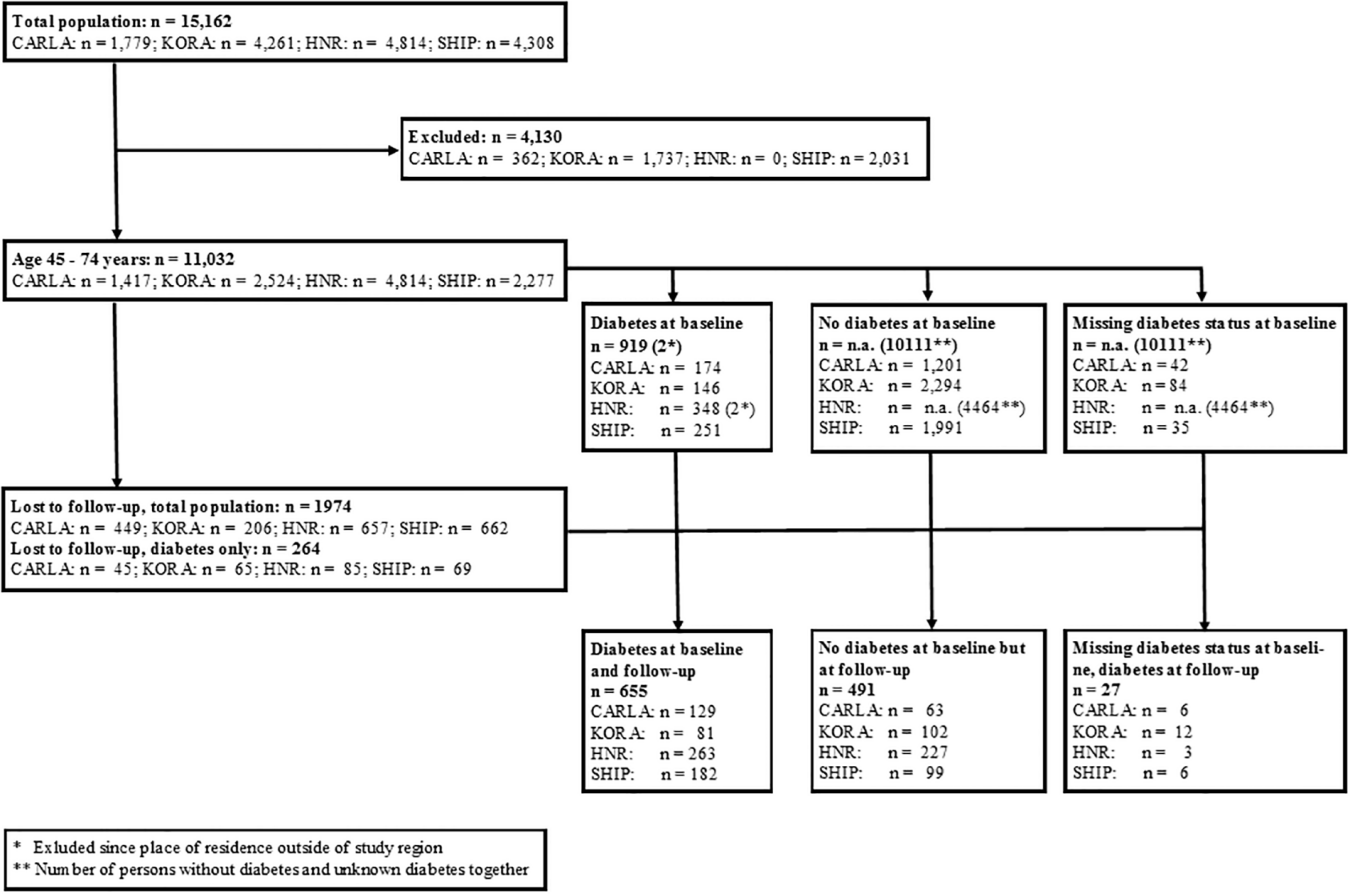
Flow-chart study population.

To increase comparability, participants’ age was limited to 45 to 74 years at baseline, corresponding to 49 to 83 years at follow up. A further inclusion criterion was having type 2 diabetes mellitus at baseline or follow-up examination (defined as self-reported physician’s diagnosis of diabetes and age at diabetes onset of at least 30 years) resulting in the final study population of 1,437 participants (Fig 1, Table 1).

#### 2.1.2 Medication

To record medication intake, participants should bring the original packages of all medications they had used during the seven days preceding the baseline or follow-up examination. According to the corresponding ATC (Anatomical Therapeutic Chemical Classification System) codes antihyperglycemic and cardiovascular medication were identified and anti-hyperglycemic medication (A10) were subdivided into all types of insulin (A10A), and oral anti-hyperglycemic agents (A10B). In accordance with Tamayo et al. and Waugh et al. [11,17] “newer” anti-hyperglycemic agents were defined as follows:

- Insulin analogues: Lispro (A10AB04, A10AC04) and combinations with Lispro (A10AD04), Aspart (A10AB05) and combinations (A10AC05, A10AD05), Glulisine (A10AB06), Glargine (A10AE04), Detemir (A10AE05);
- Newer oral anti-hyperglycemic medications: thiazolidinediones (A10BG: e.g., Rosiglitazone, Pioglitazone), glinides (A10BX: e.g., Repaglinide, Nateglinide), DPP4-inhibitors (A10BH), and combinations of thiazolidinediones or glinides with metformin or glimepiride (A10BD03-A10BD08).

#### 2.1.3 Anthropometry, laboratory, comorbidity

Furthermore, data on body mass index [kg/m^2^] (calculated from measured weight and height), systolic and diastolic blood pressure [mmHg] (mean of the second and third measurement taken by trained personal using validated automatic devices), and HbA_1c_ [%, mmol/mol] (included despite different assessment methods to consider the confounding effect in stratified analyses) were included as well as data on comorbidity (self-reported history of medically confirmed stroke or myocardial infarction), intake of cardiovascular medication [ATC C]).

#### 2.1.4 Lifestyle

Lifestyle was described using the following components: self-reported smoking status (divided into “current smokers”, i.e., ≥1 cigarette/day vs. “never-smokers” or “ex-smokers” previously smoking ≥1 cigarette/day, but quitting smoking >1 year ago), and alcohol consumption (“high-risk”: >20/40 g/day in women/men [18,19]; calculated from self-reported weekly consumption of beer, wine, and liquor [19].

#### 2.1.5 Individual sociodemographic and socioeconomic variables

Family status was approximated using the dichotomous variable “living with a partner” (yes/no). Educational level was defined based on the highest achieved schooling degree (“low”: no schooling degree; “intermediate”: junior high school attendance or secondary school certificate graduation [corresponding to at least 8 completed years of schooling]; “high”: high educational graduation [corresponding to at least 12 completed years of schooling]). Furthermore, the highest achieved level of vocational qualification was included (“low”: no vocational qualification; “intermediate”: apprenticeship, completed vocational, technical or master school; “high”: university degree; “other”: other vocational qualification). Net household income per month and household size were used to calculate equivalent income (income/household size^0,36^) as suggested in the Luxembourg Income Study and used in earlier studies of the DIAB-CORE consortium [5,11].

#### 2.1.6 Regional deprivation

Individual participant data were supplemented by the German Index of Multiple Deprivation (GIMD) already used in a number of studies [4,5]. Using data derived from official statistics (here: mostly from 2006) the index exists on municipality and district level including seven deprivation domains (income, employment, education, municipal/district revenue, social capital, environment, security) with higher values representing more deprived areas. In this study the index was used on district level.

#### 2.1.7 Regional health care services

Indicators of regional health care services on district level were collected from various sources between 1997 and 2010 (Table 2): the number of hospital beds, physicians, internists per 100,000 inhabitants from official statistics [20,21], the number of diabetologists/100,000 inhabitants from the German Diabetes Association (DDG), and the number of diabetes disease management program (DMP) participants/100,000 inhabitants (federal state level) derived from yearly quality reports of the Federal Association of Statutory Health Insurance Physicians (Kassenärztliche Bundesvereinigung) [22].

**Table 2:** Indicators of health care services included in the analyses.

Regional indicator	Unit	Source
Hospital bed density (years 1997–2010)	Hospital beds/100,000 inhabitants	Federal Statistical Office {Federal Statistical Office, statistical offices of the German states #1153}
Physician density (years 1997–2010)	Physicians/100,000 inhabitants	Federal Institute for Building, Urban Affairs and Spatial Research {Federal Institute for Building, Urban Affairs and Spatial Research #1154}
Internist density (years 1997–2010)	Internists/100,000 inhabitants	Federal Institute for Building, Urban Affairs and Spatial Research {Federal Institute for Building, Urban Affairs and Spatial Research #1154}
Diabetologist density (years 2000–2010)	Diabetologists/100,000 inhabitants	German Diabetes Association (DDG), Federal Statistical Office {Federal Statistical Office, statistical offices of the German states #1153}{
Diabetes disease management program (DMP) participants (federal state level) (years 2004–2010)	DMP participants/100,000 inhabitants	Federal Association of Statutory Health Insurance Physicians {Federal Association of Statutory Health Insurance Physicians #1155}

### 2.2 Statistical analysis

Individual, regional deprivation and health care services data were merged based on official district keys. The description was stratified both by examination (baseline, follow up) and by regional study. Means and standard deviations (SDs) were used for the description of continuous variables. Categorical variables were described by numbers and proportions. Furthermore, proportions of treatment with anti-hyperglycemic pharmaceuticals were determined.

The association between study region and anti-hyperglycemic treatment was analyzed for two dependent outcome variables: I. intake of any anti-hyperglycemic medication in the total sample, II. intake of newer anti-hyperglycemic medication among participants with any anti-hyperglycemic treatment. Since the prevalence of medication intake was the outcome of interest both baseline and follow-up data were analyzed cross-sectionally. In accordance with Zou et al.[23] prevalence ratios (PRs) for the intake of any/newer anti-hyperglycemic medication were estimated by multivariable Poisson regression models with a robust error variance using log link function. This methodological approach was preferred because of the high prevalence of all outcomes resulting in overestimations of the true effects when odds ratios from logistic regression models would be computed instead [24].

To account for the variation between baseline and follow-up examinations, a mixed model approach was applied for outcome I (any anti-hyperglycemic medication) using “person” as random effect [25]. Districts and federal states were not considered as random effects because the number of districts (n = 11) and federal states (n = 4) in the study regions was considerably low. Since a poisson model was the model of choice, mixed effects poisson models (PROC GLIMMIX) were calculated. Because of differences in the study periods affecting the availability of newer anti-hyperglycemic medication, the association for outcome II was examined solely among participants of the follow-up examination (N = 894). Hence a standard poisson model with robust error variance was calculated.

For both outcomes, six basis models were fitted.

- Model 1: crude model
- Model 2: adjusted for individual variables (age, sex, year of examination, diabetes duration)
- Model 3: additionally adjusted for variables of anthropometry and comorbidity
- Model 4: additionally adjusted for lifestyle and individual sociodemographic and socioeconomic variables
- Model 5: additionally adjusted for regional deprivation (GIMD)
- Model 6: additionally adjusted for single indicators of health care services

Because of the high correlation between the regional variables it was not possible to adjust for all regional health care structure variables in a joint model. Furthermore, the huge number of regression models did not allow showing PRs for each variable included in the regression models. For ease of clarity only PRs of the study regions were presented in the resulting tables.

All analyses were performed using SAS statistical software version 9.3 (SAS Institute Inc., Cary, NC, USA).

## 3. Results

### 3.1 Study population

Descriptive data of the study population and anti-hyperglycemic treatment in total as well as stratified by study and examination are summarized in Tables 3 and 4.

**Table 3:** Description of variables.

ID	abbreviation	name of variable [unit]	nature of variable	missing at baseline	missing at follow-up
**Individual variables**					
1	Sex	Sex (male, female)	categorial	0	0
2	AgeE	Age at examination [years]	continuous	0	0
3	AgeD	Age at diagnosis of diabetes [years]	continuous	0	0
4	DD	Diabetes duration [years]	continuous	0	0
**Anthropometry/Comorbidity variables**					
5	HbA_1c_^1^	HbA_1c_ [%]	continuous	10	29
6	HbA_1c_^2^	HbA_1c_ [mmol/mol]	continuous	10	29
7	BMI	Body mass index [kg/m^2^]	continuous	2	10
8	SBP	Systolic blood pressure [mmHg]	continuous	3	3
9	DBP	Diastolic blood pressure [mmHg]	continuous	2	3
10	CVDM	Medication of the cardiovascular system (ATC C)	continuous	0	0
11	Stroke	Previous medically confirmed stroke (yes, no)	categorial	4	4
12	MI	Previous medically confirmed myocardial infarction	categorial	7	4
**Lifestyle variables**					
13	Smoke (No, Ex, Curr)	Smoking status (no smoker, former smoker, current smoker)	categorial	0	6
14	Alc	High-risk alcohol consumption	categorial	16	70
**Individual sociodemographic/socioeconomic variables**					
15	Partner	Living with a partner (yes, no)	categorial	0	3
16	School (Low, Int, High)	School degree (low, intermediate, high)	categorial	0	4
17	Voc (Low, Int, High,Other)	Vocational qualification (low, intermediate, high, other)	categorial	1	2
18	EQHI	Equivalent household income [€]	continuous	44	100
**Ecological variables**					
19	GIMD	Regional deprivation [GIMD-Score]	continuous	0	0
**Indicators of health care structure**					
20	Beds	Hospital beds/100,000 inhabitants	continuous	0	0
21	Phys	Physicians/100,000 inhabitants	continuous	0	0
22	Int	Internists/100,000 inhabitants	continuous	0	0
23	Diab	Diabetologists/100,000 inhabitants	continuous	227	0
24	DMP	DMP participants/100,000 inhabitants	continuous	919	98
**Anti-hyperglycemic treatment (according to ATC-Codes)**					
25	A10	Any anti-hyperglycemic medication (oral or insulin) (ATC A10)	categorial	0	0
26	A10B	Total oral anti-hyperglycemic treatment (ATC A10B)	categorial	0	0
27	A10BA	Metformin (ATC A10BA)	categorial	0	0
28	A10BB	Sulfonylureas(ATC A10BB)	categorial	0	0
29	A10BD	Combinations of oral anti-hyperglycemic medication (Metformin Glitazones/ DPP4-inhibitors) (ATC A10BD)	categorial	0	0
30	A10BF	α-Glucosidaseinhibitors (Acarbose/Miglitol) (ATC A10BF)	categorial	0	0
31	A10BG	Thiazolidinediones (Glitazone) (ATC A10BG)	categorial	0	0
32	A10BH	DPP4-inhibitors (ATC A10BH)	categorial	0	0
33	A10BX	Glinide (ATC A10BX)	categorial	0	0
34	A10A	Treatment with any insulin (ATC A10A)	categorial	0	0
35	Unknown	Unknown medication	categorial	0	0
**Newer anti-hyperglycemic treatment among participants with any anti-hyperglycemic medication**					
36	NewMedi	All newer medication (insulin analogue and/or oral combinations)	categorial	0	0
37	NewOralM	Any newer oral medication	categorial	0	0
38	NewInsM	Any (newer) insulin analogues	categorial	0	0

**Table 4:** Characteristics and patterns of anti-hyperglycemic treatment of participants with type 2 diabetes mellitus in the CARLA, KORA, HNR and SHIP studies.

		Population at baseline examination					Population at follow-up examination				
		Total (n = 919)	CARLA (n = 174)	KORA S4 (n = 146)	HNR (n = 348)	SHIP 0 (n = 251)	Total (n = 1,173)	CARLA F1 (n = 198)	KORA F4 (n = 195)	HNR (n = 493)	SHIP 1 (n = 287)
ID	**Individual variables**										
1	Sex, male n (%)	514 (55.9)	96 (55.2)	80 (54.8)	210 (60.3)	128 (51.0)	685 (58.4)	119 (60.1)	110 (56.4)	302 (61.3)	154 (53.7)
2	AgeE (mean, SD)	63.1 (7.2)	63.5 (7.3)	63.3 (6.7)	63.0 (7.2)	62.8 (7.5)	66.9 (7.4)	66.2 (7.3)	68.5 (7.3)	66.8 (7.1)	66.6 (7.9)
3	AgeD (mean, SD)	54.4 (9.3)	54.6 (9.5)	54.6 (9.2)	55.0 (9.5)	53.3 (8.9)	58.1 (10.0)	57.3 (10.0)	59.9 (9.8)	58.9 (9.8)	56.2 (10.0)
4	DD (mean, SD)	8.7 (7.6)	8.8 (7.6)	8.6 (7.2)	8.0 (8.1)	9.6 (7.1)	8.8 (8.1)	8.9 (8.2)	8.6 (8.1)	8.0 (8.0)	10.3 (8.0)
	**Anthropometry/Comorbidity variables**										
5	HbA_1c_^1^(mean,SD)	^a^	7.0 (1.4)	7.1 (1.4)	7.0 (1.5)	7.2 (1.5)	^a^	6.8 (1.0)	6.8 (1.0)	6.8 (1.2)	6.9 (1.3)
6	HbA_1c_^2^(mean,SD)	^a^	53 (15)	54 (15)	53 (16)	56 (16)	^a^	51 (11)	51 (11)	51 (13)	52 (14)
7	BMI (mean, SD)	30.9 (5.2)	30.9 (4.9)	32.0 (5.5)	30.4 (5.3)	31.0 (5.1)	31.1 (5.4)	31.3 (5.3)	31.4 (5.5)	30.7 (5.4)	31.3 (5.3)
8	SBP (mean, SD)	145.9(22.3)	150.8(23.0)	144.2(21.6)	141.2(22.2)	150.1 (20.9)	130.0 (21.3)	140.5 (20.5)	132.9 (20.5)	138.7(20.8)	142.4(22.5)
9	DBP (mean, SD)	82.9 (11.4)	85.1 (12.3)	83.1 (12.0)	81.1 (10.7)	84.0 (11.1)	77.7 (10.9)	78.5 (10.3)	74.0 (10.4)	77.5 (10.6)	79.8(11.5)
10	CVDM n (%)	701(76.3)	148(85.1)	103(70.6)	250(71.8)	200(79.7)	1011(86.2)	181(91.4)	163(83.6)	412(83.6)	255(88.9)
11	Stroke n (%)	57(6.2)	13(7.5)	5(3.4)	23(6.7)	16(6.4)	85(7.3)	15(7.7)	12(6.2)	37(7.5)	21(7.4)
12	MI n (%)	94(10.3)	12(7.0)	15(10.3)	37(10.7)	30(12.2)	123(10.5)	18(9.2)	21(10.8)	48(9.7)	36(12.6)
	**Lifestyle variables**										
13	Smoke										
	No	439 (47.8)	82(47.1)	65(44.5)	151(43.4)	141(56.2)	464(39.8)	75(38.3)	75(38.5)	195(39.6)	119(41.9)
	Ex	339 (36.9)	64(36.8)	63(43.2)	133(38.2)	79(31.5)	547(46.9)	86(43.9)	98(50.3)	227(46.1)	136(47.9)
	Curr	141 (15.3)	28(16.1)	18(12.3)	64(18.4)	31(12.4)	156(13.4)	35(17.9)	22(11.3)	70(14.2)	29(10.2)
14	Alc n (%)	58(6.4)	7(4.0)	21(14.5)	18(5.4)	12(4.8)	75(6.8)	11(5.6)	23(11.8)	30(6.3)	11(4.7)
	**Individual sociodemographic/socioeconomic variables**										
15	Partner n (%)	702(76.4)	126(72.4)	111(76.0)	279(80.2)	186(74.1)	905(77.4)	145(74.0)	147(75.4)	401(81.5)	212(73.9)
16	School n (%)										
	Low	40(4.4)	12(6.9)	6(4.1)	10(2.9)	12(4.8)	37(3.2)	11(5.6)	5(2.6)	11(2.2)	10(3.5)
	Int	759(82.6)	129(74.1)	126(86.3)	285(81.9)	219(87.3)	959(82.0)	144(72.7)	164(84.1)	400(81.6)	251(87.8)
	High	120(13.1)	33(19.0)	14(9.6)	53(15.2)	20(8.0)	173(14.8)	43(21.7)	26(13.3)	79(16.1)	25(8.7)
17	Voc n (%)										
	Low	180(19.6)	13(7.5)	45(30.8)	59(17.0)	63(25.1)	170(14.5)	9(4.6)	33(16.9)	68(13.8)	60(21.0)
	Int	620(67.5)	140(80.5)	92(63.0)	239(68.9)	149(59.4)	841(71.8)	163(82.3)	140(71.8)	355(72.2)	183(64.0)
	High	104(11.3)	11(6.3)	9(6.2)	49(14.1)	35(13.9)	146(12.5)	14(7.1)	22(11.3)	69(14.0)	41(14.3)
	Other	14(1.5)	10(5.8)	0(0.0)	0(0.0)	4(1.6)	14(1.2)	12(6.1)	0(0.0)	0(0.0)	2(0.7)
18	EQHI(mean, SD)	1434.2 (841.7)	1388.3 (720.1)	1397.5 (763.0)	1755.5 (1001.8)	1043.0 (503.0)	1589.0 (776.2)	1350.1 (606.0)	1531.0 (664.8)	1810.9 (888.0)	1372.4 (590.5)
	**Ecological variables**										
19	GIMD(mean,SD)	32.6 (12.0)	35.9 (0.0)	18.5 (12.8)	26.8 (4.1)	46.7 (6.2)	31.6 (12.1)	35.9 (0.0)	18.0 (12.7)	26.3 (4.1)	47.0 (6.2)
	**Indicators of health care structure**										
20	Beds (mean, SD)	839 (415.8)	698.5(31.3)	700.5(504.8)	824.3(162.8)	1040.1 (619.3)	809.1 (411.6)	736.0(11.2)	611.7(470.3)	799.6(181.0)	1009.8 (645.8)
21	Phys (mean, SD)	171 (42.6)	223.3 (2.0)	174.2 (53.2)	147.7 (6.8)	165.1 (47.4)	179.2 (47.3)	235.3 (4.1)	189.1 (59.7)	154.1 (6.4)	176.8 (57.6)
22	Int (mean, SD)	25.0 (6.4)	30.4 (0.7)	22.2 (8.8)	27.8 (3.2)	18.9 (4.1)	27.5 (7.0)	35.0 (1.5)	24.8 (9.6)	28.3 (3.3)	22.8 (7.2)
23	Diab (mean, SD)	4.3 (2.2)	3.6 (0.6)	2.0 (1.4)	5.2 (1.8)	6.5 (3.9)	5.7 (3.2)	4.3 (0.0)	2.8 (1.8)	7.4 (2.2)	5.9 (4.5)
24	DMP^b^(mean,SD)	--------	--------	--------	--------	--------	4452.1 (1514.4)	7092.9 (417.2)	3544.7 (261.6)	4385.3 (752.9)	2796.0 (630.3)
	**Anti-hyperglycemic treatment (according to ATC-Codes)**^c^										
25	A10 n (%)	685(74.5)	137(78.7)	121(82.9)	242(69.5)	185(73.7)	894(76.2)	153(77.3)	161(82.6)	356(72.2)	224(78.1)
26	A10B n (%)	544(59.2)	112(64.4)	100(68.5)	192(55.2)	140(55.8)	746(63.6)	128(64.7)	139(71.3)	302(61.3)	177(61.7)
27	A10BA n (%)	322(35.0)	63(36.2)	55(37.7)	131(37.6)	73(29.1)	538(45.9)	83(41.9)	95(48.7)	234(47.5)	126(43.9)
28	A10BB n (%)	306(33.3)	58(33.3)	62(42.5)	95(27.3)	91(36.3)	305(26.0)	54(27.3)	59(30.3)	114(23.1)	78(27.2)
29	A10BD n (%)	0(0.0)	0(0.0)	0(0.0)	0(0.0)	0(0.0)	23(2.0)	5(2.5)	7(3.6)	8(1.6)	3(1.1)
30	A10BF n (%)	52(5.7)	11(6.3)	14(9.6)	18(5.2)	9(3.6)	19(1.6)	8(4.0)	3(1.5)	4(0.8)	4(1.4)
31	A10BG n (%)	14(1.5)	7(4.0)	0(0.0)	7(2.0)	0(0.0)	34(2.9)	3(1.5)	11(5.6)	11(2.2)	9(3.1)
32	A10BH n (%)	0(0.0)	0(0.0)	0(0.0)	0(0.0)	0(0.0)	4(0.3)	1(0.5)	1(0.5)	2(0.4)	0(0.0)
33	A10BX n (%)	27(2.9)	9(5.2)	1(0.7)	15(4.3)	2(0.8)	44(3.8)	8(4.0)	6(3.1)	22(4.5)	8(2.8)
34	A10A) n (%)	233(25.4)	55(31.6)	39(26.7)	68(19.5)	71(28.3)	279(23.8)	54(27.3)	47(24.1)	90(18.3)	88(30.7)
35	Unknown n (%)	3(0.3)	0(0.0)	0(0.0)	3(0.9)	0(0.0)	4(0.3)	0(0.0)	0(0.0)	4(0.8)	0(0.0)
	**Newer anti-hyperglycemic treatment among participants with any anti-hyperglycemic medication (n_baseline_ = 685, n_follow up_ = 894)** ^c^^,^^d^										
36	NewMedi n (%)	100(14.6)	34(24.8)	6(5.0)	48(19.8)	12(6.5)	207(23.2)	43(28.1)	48(29.8)	71(19.9)	45(20.1)
37	NewOralM n (%)	40(5.8)	16(11.7)	1(0.8)	21(8.7)	2(1.1)	101(11.3)	16(10.5)	24(14.9)	41(11.5)	20(8.9)
38	NewInsM n (%)	64(9.3)	21(15.3)	5(4.1)	28(11.6)	10(5.4)	119(13.3)	29(19.0)	29(18.0)	34(9.6)	27(12.1)

Data are described as proportion or mean (SD)

^a^ Not summable because of study differences in blood samples, measurement methods and standard operating procedures.

^b^ Because disease management programs (DMPs) for diabetes have been implemented only in 2002, this analysis was restricted to follow up observations.

^c^ Multiple entries possible

^d^ Percentages based on individuals with any anti-hyperglycemic treatment (ATC: A10).

### 3.2. Determinants of any anti-hyperglycemic medication in the total study population

Table 5 summarizes pairwise PRs for any anti-hyperglycemic medication. According to the results of the crude model 1, treatment patterns varied by study region. KORA participants were more likely to receive any anti-hyperglycemic medication than participants from all other studies, while least prescriptions were found in HNR. These differences were independent from all individual variables (models 2–4). The regional differences persisted after adjustment for regional deprivation (model 5). Further adjustment for single indicators of health care structure (model 6) resulted in mostly minor variations of PRs. The statistically significant difference in the medication prevalence between KORA vs. HNR reported in models 1 to 5 remained in the same order of magnitude with PRs ranging between 1.12 after adjustment for DMP participants/100,000 inhabitants and 1.15 after adjustment for diabetologists/100,000 inhabitants.

**Table 5:** Multivariable prevalence ratios of anti-hyperglycemic medication.

	KORA vs. HNR	CARLA vs. HNR	SHIP vs. HNR
Model 1	1.16* (1.08–1.25)	1.10* (1.01–1.18)	1.07 (1.00–1.15)
**Models 2–4 adjusted for individual variables**			
Model 2	1.16* (1.08–1.24)	1.08* (1.00–1.17)	1.06 (0.98–1.14)
Model 3	1.16* (1.08–1.24)	1.08 (1.00–1.16)	1.05 (0.98–1.13)
Model 4	1.14* (1.06–1.23)	1.07 (0.99–1.16)	1.03 (0.94–1.12)
**Model 5 adjusted for individual variables (model 4) + regional deprivation (GIMD)**			
GIMD	1.14* (1.06–1.23)	1.07 (0.98–1.16)	1.02 (0.91–1.15)
**Model 6 adjusted for individual variables + regional deprivation (GIMD; model 5) + single indicators of health care structure**			
Hospital beds/100,000 inhabitants	1.14* (1.06–1.23)	1.08 (0.99–1.18)	1.04 (0.93–1.17)
Physicians/100,000 inhabitants	1.14* (1.5–1.25)	1.08 (0.98–1.18)	1.04 (0.93–1.17)
Internists/100,000 inhabitants	1.14* (1.06–1.23)	1.08 (0.99–1.18)	1.01 (0.87–1.16)
Diabetologists/100,000 inhabitants	1.15* (1.06–1.25)	1.10 (1.00–1.21)	1.08 (0.96–1.22)
DMP participants/100,000 inhabitants^a^	1.12* (1.01–1.24)	1.14 (0.93–1.39)	1.00 (0.82–1.22)

Prevalence ratios and 95% CIs derived from mixed effects Poisson models with robust error variance

* p<0.05

^a^ Because disease management programs (DMPs) for diabetes have been implemented only in 2002, this analysis was restricted to follow up observations.

Model 1 crude model (N = 1,437, 2,092 observations)

Model 2: adjusted for age, sex, year of examination, and diabetes duration (N = 1,437, 2,092 observations)

Model 3: Model 2 + additional adjustment for HbA_1C_, body mass index, systolic blood pressure, diastolic blood pressure, medication of the cardiovascular system, self-reported medically confirmed myocardial infarction, and self-reported medically confirmedstroke (N = 1,408, 2,027 observations)

Model 4: Model 3 + additional adjustment for smoking status, alcohol consumption, living with a partner, school degree, vocational qualification, and equivalent household income (N = 1,322, 1,825 observations)

Models 5: Model 4 + additional adjustment for regional deprivation (GIMD; N = 1,322, 1,825 observations),

Model 6: Model 5 + additional single adjustment for hospital beds/100,000 inhabitants (N = 1,322, 1,825 observations), physicians per 100,000 inhabitants (N = 1,322, 1,825 observations), internists per 100,000 inhabitants (N = 1,322, 1,825 observations), diabetologists/100,000 inhabitants (N = 1,212, 1,619 observations), or DMP participants/100,000 inhabitants (N = 923, 923 observations)

Regarding the effect of other independent variables, the prevalence of receiving anti-hyperglycemic medication increased significantly with increasing diabetes duration, HbA_1c_ and intake of cardiovascular medication in all regression models (all p<0.001; data not shown). Furthermore, increasing diastolic blood pressure was predominantly associated with decreased prevalence of anti-hyperglycemic medication.

### 3.3. Determinants of newer anti-hyperglycemic medication among people with any anti-hyperglycemic treatment

As shown in Table 6, regional differences in the prevalence of receiving newer anti-hyperglycemic medication were more pronounced than regarding any anti-hyperglycemic medication. According to the crude model 1, regional differences varied between 1% and 49% with significantly higher proportions in KORA and CARLA compared with HNR. After adjustment for individual basis variables (model 2), the difference between KORA vs. HNR increased while the difference between CARLA vs. HNR decreased and was no longer statistically significant. Adjustments for further individual variables (models 3–4) changed PRs only marginally. Additional adjustment for regional deprivation (model 5) increased regional differences in the prevalence of receiving newer anti-hyperglycemic medication between KORA vs. HNR, while the difference between CARLA vs. HNR decreased (not statistically significant). After further inclusion of single indicators of health care structure, significant differences in the regional prevalence of newer anti-hyperglycemic medication between KORA and HNR mainly persisted while the direction of the change in PRs for CARLA or SHIP vs. HNR was inconsistent and depended on the included indicator of health care structures.

**Table 6:** Multivariable prevalence ratios of new antihyperglycemic medication among participants with any anti-hyperglycemic medication.

	KORA vs. HNR	CARLA vs. HNR	SHIP vs. HNR
Model 1	1.49* (1.09–2.05)	1.41* (1.02–1.96)	1.01 (0.72–1.41)
**Models 2–4 adjusted for individual variables**			
Model 2	1.57* (1.16–2.14)	1.29 (0.91–1.82)	1.04 (0.57–1.90)
Model 3	1.61* (1.18–2.19)	1.30 (0.91–1.86)	1.18 (0.64–2.18)
Model 4	1.54* (1.12–2.13)	1.20 (0.82–1.76)	1.30 (0.61–2.80)
**Model 5 adjusted for individual variables (model 4) + regional deprivation (GIMD)**			
GIMD Score	1.67* (1.20–2.32)	1.05 (0.70–1.57)	0.99 (0.43–2.31)
**Model 6 adjusted for individual variables + regional deprivation (GIMD; model 5) + single indicators of health care structure**			
Hospital beds/100,000 inhabitants	1.66* (1.20–2.31)	1.10 (0.72–1.67)	1.08 (0.45–2.58)
Physicians/100,000 inhabitants	1.33 (0.87–2.02)	0.88 (0.55–1.39)	1.18 (0.51–2.75)
Internists/100,000 inhabitants	1.56* (1.13–2.16)	0.99 (0.65–1.49)	1.76 (0.70–4.41)
Diabetologists/100,000 inhabitants	1.68* (1.18–2.40)	1.06 (0.67–1.67)	1.00 (0.43–2.30)
DMP participants/100,000 inhabitants^a^	2.03* (1.35–3.05)	0.51 (0.20–1.30)	1.97 (0.75–5.15)

Prevalence ratios and 95% CIs derived from Poisson models with robust error variance

* p<0.05

^a^ Because disease management programs (DMPs) for diabetes have been implemented only in 2002, this analysis was restricted to follow up observations.

Model 1: crude model (N = 894)

Model 2: adjusted for age, sex, year of examination and diabetes duration (N = 894)

Model 3: Model 2 + additional adjustment for HbA_1C_, body mass index, systolic blood pressure, diastolic blood pressure, medication of the cardiovascular system, self-reported medically confirmedmyocardial infarction, and self-reported medically confirmedstroke (N = 863)

Model 4: Model 3 + additional adjustment for smoking, alcohol consumption, living with a partner, school degree, vocational qualification, equivalent household income (N = 750)

Models 5: Model 4 + additional adjustment for regional deprivation (N = 750)

Model 6: Model 5 + additional single adjustment for hospital beds/100,000 inhabitants (N = 750), physicians per 100,000 inhabitants (N = 750), internists per 100,000 inhabitants (N = 750), diabetologists/100,000 inhabitants (N = 750), or DMP participants/100,000 inhabitants (N = 696)

Regarding the effect of other independent variables, the prevalence of receiving newer anti-hyperglycemic medication was higher with increasing diabetes duration and HbA_1c_ in most regression models, but lower with increasing age (data not shown).

## 4. Discussion

### 4.1 Key results

Analyses of medication data from four longitudinal, population-based German studies partly showed considerable regional differences in anti-hyperglycemic medication with differences in newer medication prevalence of up to 49%. Regarding any anti-hyperglycemic medication, neither adjustment for individual variables, regional deprivation nor indicators of health care services could completely explain regional differences. Compared with any anti-hyperglycemic medication, regional differences in the prevalence of receiving any newer anti-hyperglycemic medication were more pronounced. The prevalence of receiving anti-hyperglycemic and newer medication was thereby highest in the south and lowest in the west. After adjustment for individual variables statistically significant regional differences persisted only for KORA (south) vs. HNR (west). Despite extensive adjustment regional differences in the prevalence of any as well as newer anti-hyperglycemic medication mainly remained indicating associations with further influencing factors not captured in the present analyses.

### 4.2 Comparison with other studies

Although the interest in health care differences and the underlying causes is high and further growing, corresponding literature remains scarce. Especially individual and regional associations have rarely been analyzed simultaneously. Compared with previous results by Tamayo et al. adjusted for individual variables [11], regional differences in any anti-hyperglycemic medication between KORA (south) and HNR (west) were confirmed, and further differences between CARLA (east) vs. HNR (west) appeared. However, there was no consistent difference between the studies from western (KORA, HNR) and the studies from eastern Germany (SHIP, CARLA) in contrast to some other previously reported health outcomes [26]. The current findings support the results by Tamayo et al. suggesting that individual variables explain regional differences inadequately. Although regional deprivation could partly explain these differences in type 2 diabetes mellitus prevalence [4–6], regional deprivation seems not to be of significant importance in the explanation of differences in antihyperglycemic medication in this study either.

Comparisons with studies from other countries are limited because of differences in regional structures and health care systems (especially regarding the reimbursement of anti-hyperglycemic medication) [27]. The association between regional deprivation and worse diabetes outcomes reported in the review by Grintsova et al. [8], was not found for anti-hyperglycemic medication in this study. However, it is unknown if a higher proportion of medication use implicate a worse or better quality of treatment or even a mixture of both masking potential apparent differences in diabetes care as a consequence. In Belgium, Wens et al. demonstrated regional differences in the first utilization of anti-hyperglycemic medication (sulphonylureas vs. biguanides) independent of body mass index, HbA_1c_, serum cholesterol and triglycerides [28]. In Germany, the utilization of the biguanide metformin is still recommended as first choice medication in the national diabetes guidelines {Bundesärztekammer (BÄK), Kassenärztliche Bundesvereinigung (KBV), Arbeitsgemeinschaft der Wissenschaftlichen Medizinischen Fachgesellschaften (AWMF), Arzneimittelkommission der deutschen Ärzteschaft (AkdÄ), Deutsche Diabetes Gesellschaft (DDG), Deutsche Gesellschaft für Allgemeinmedizin und Familienmedizin (DEGAM), Deutsche Gesellschaft für Innere Medizin (DGIM), Verband der Diabetesberatungs- und Schulungsberufe Deutschland [29]. Correspondingly, metformin utilization at follow-up was consistently higher in all current studies than utilization of sulfonylureas. Moreover, analyses of the variability of prescribing for diabetes and secondary preventative therapies revealed large differences between eight Irish health board regions not explainable by differences in the distribution of age and gender [30]. However, socioeconomic differences and differences in health care services between the regions were not considered in the respective study.

### 4.3 Implications

Summarizing the current results neither individual variables including individual socio economic status nor regional variables describing regional deprivation and health care services were able to sufficiently explain regional differences in any and newer anti-hyperglycemic treatment. To understand the underlying causes, future studies may also consider the influence of individual health behavior (e.g., dietary behavior, diabetes knowledge, health literacy, attitudes, wishes, and compliance regarding diabetes therapy), physicians′ attitudes (e.g., regarding continual medical education and prescriptions), physician-patient interactions and reimbursement, the quality and utilization of regional health care services, and the prevalence of chronic stress and mental health problems. Further, possible differences in the detection of diabetes have to be taken into account. In addition, analyses of regional differences based on small-area data, e.g. at the municipal level, might be more informative. Another point of interest is the association between regional differences in anti-hyperglycemic treatment and long-term diabetes outcomes.

Interestingly, regional differences as reflected by PRs seemed to be more pronounced regarding the prevalence of newer anti-hyperglycemic medication compared with any anti-hyperglycemic medication (in line with the study by Tamayo et al. [11]). The underlying causes are not known to date. Maybe, differences in the budgets of the treating physicians depending on the regionally organized Associations of Statutory Health Insurance Physicians (Kassenärztliche Vereinigungen) and differences in health insurance membership of the treated patients (selective contracts, statutory vs. private health insurance) are of importance in this context.

### 4.4 Strengths and limitations

The strengths of our study are the utilization of data from four German regional population-based studies with high comparability regarding sampling procedure, study design, and assessment tools. For the first time in Germany and elsewhere, individual socio economic status, lifestyle factors, regional deprivation, and differences in health care services were considered together trying to explain differences in antihyperglycemic medication in general and stratified by type of medication in a large sample of participants.

However, this study is limited by differences in the periods of data collection between the studies. Although response rates were similar (baseline 56%-69%, follow-up 80%-90%), differences in nonresponse may have biased the regional analyses. The use of districts as regional reference may have led to distortion of the results because of heterogeneous geographical sizes and numbers of inhabitants. Another limitation is that individual data on health insurance status (statutorily or privately insured) were not available for all studies. Furthermore, the results of model 6 should be interpreted with caution in the context of an increased uncertainty of estimates and standard errors due to high collinearity of regional variables with study region. To ensure a uniform procedure it was decided to limit the analysis of new anti-hyperglycemic medication to follow-up data, although the time of the baseline examination of some studies overlapped with the study period at follow-up of others. In addition, PRs regarding new anti-hyperglycemic medication were often not statistically significant despite partly considerably regional differences due to low statistical power.

In conclusion, for the first time, regional differences in any and newer anti-hyperglycemic treatment have been demonstrated based on data from four regional population-based studies in Germany. Because neither comprehensive individual variables nor regional deprivation and health care services were able to sufficiently explain regional differences, further research is needed to understand the underlying causes, assess implications for type 2 diabetes mellitus outcomes, and plan interventions for deprived target groups.
